# Flammable Substances in Korea Considering the Domino Effect: Assessment of Safety Distance

**DOI:** 10.3390/ijerph16060969

**Published:** 2019-03-18

**Authors:** Hyo Eun Lee, Seok J. Yoon, Jong-Ryeul Sohn, Da-An Huh, Bong Woo Lee, Kyong Whan Moon

**Affiliations:** 1 Department of Health Science, Korea University, Anam-ro 145, Seongbuk-gu, Seoul 02841, Korea; chokbab@naver.com (H.E.L.); ehslab@naver.com (S.J.Y.); sohn1956@korea.ac.kr (J.-R.S.); black1388@korea.ac.kr (D.-A.H.); 2 Korea Fire Institute, Jisam-ro 331 Giheung-gu, Youngin-Si, Gyeonggi-do 17088, Korea; silicones@hanmail.net

**Keywords:** domino explosion, safety distance, vapor cloud explosion, BTX, Areal Location of Hazardous Atmosphere, Process Hazard Analysis Software Tool

## Abstract

Benzene, toluene, and xylene (BTX) are flammable substances used in a wide range of raw materials and products. Chemical accidents caused by flammable substances are different from leakage accidents of toxic materials. Initial explosions and fires may cause secondary or tertiary explosions, or fires with nearby flammable materials. This is called the domino effect. In cases of leakage accidents, it is possible to prevent accidents through early control of the leakage to the outside or by bypassing, but it is difficult to cope with explosions because they occur instantaneously. To prevent explosions due to the domino effect, a safety distance must be set. Safety distances vary widely by country. In the case of the United States (US) or the European Union (EU), safety distances are set in various ways depending on the chemical industry and the amount of flammable substances being handled. However, countries such as Korea, Taiwan, and Dubai have comprehensive regulation, and the safety distances are small. In this study, we simulated the range of overpressure at which other chemical equipment could explode when an explosion occurs in a flammable BTX storage tank. There are three types of analysis methods of vapor cloud explosion. PHAST (Process Hazard Analysis Software Tool) and ALOHA (Areal Location of Hazardous Atmosphere) were selected to model explosions using three methods (trinitrotoluene equivalence method, the Netherlands Organization multi-energy method, and Baker-Strehlow-Tang method). The results indicated that the safety distances in the US and EU showed low probability of a domino effect, but those in Korea, Dubai, and Taiwan could lead to a secondary explosions. Therefore, it is necessary to propose a reasonable method to determine safety distances considering the amount and physicochemical characteristics of the flammable substances being used.

## 1. Introduction

Chemicals and related industries are the driving force of Korea’s development. Through economic development, peoples’ lives were enriched and Korea became a more advanced country [1]. Along with the growth in the chemical industry, many chemical accidents have occurred and various laws were enacted. For example, the Chemicals Control Act was created in 2012 following the Gumi hydrogen fluoride accident in South Korea. In 1976, the Seveso 2 Directive was issued following the dioxin leak accident in Seveso, Italy. In 1989, process safety management was introduced because of a petrochemical explosion at the Pasadena Phillips plant in the United States (US).

Chemical accidents can be divided into toxic chemical leaks and explosions caused by fires. Depending on whether the chemical itself is toxic or flammable, the mechanism of the chemical accident is different. If a toxic substance is leaked, then it is important to implement initial coping efforts and treatment after spreading or leakage into the surrounding environment. However, if an explosion occurs because of a fire, then it may cause damage to nearby chemical facilities [2].

Flammable substances can lead to accidents which are greater in scope than the original accident if a nearby chemical facility is damaged, owing to physical effects (overpressure and radiant heat) or if the affected chemical facility fails or explodes. This is called the domino effect [3]. 

In a previous study, from 1969 to 1998, 207 chemical accidents occurred mainly in the United States and Europe. Of these, 114 chemical accidents, 55%, caused a domino accident. Notably, 80 chemical accidents caused a second accident, and 34 caused more than three accidents. These statistics show that in the event of an explosion, more than half of the explosions do not end with the first accident and lead to subsequent accidents. [4].

A recent accident caused by the domino effect was the Tianjin Port explosion in China on 12 August 2015. The accident caused 165 deaths, 798 injuries, and an economic loss of about 1 billion dollars. Nitrocellulose (C_12_H_16_N_4_O_18_) gas generated by the fire in the warehouse destroyed the container, and various chemicals (furfuryl alcohol, ammonium nitrate, etc.) in the vicinity were rapidly decomposed at high temperature.

A second explosion occurred in a container 20 m from the first explosion. Six large fires and dozens of small fires occurred. This fire could not be suppressed for two days, and it was not suppressed until everything was burned. China then set the safety distance guidelines at the chemical plant to 1000 m [5]. Tianjin Port is a trade port where chemical substances are stored. In Korea, Busan Port and Incheon Port are also representative ports where chemicals are imported. There is a possibility that the same accident will occur in ports where chemicals are imported. In addition to the ports, domino explosions may occur at any time inside factories.

Countries worldwide are regulating the safety distances between chemical facilities. The US EPA (Environmental Protection Agency) Separation Distance Guidelines [6] and the EU (European Union) Seveso Directive also require regulations on the placement of plants considering safety distances and domino effects [7]. In particular, the safety distance is set in consideration of the characteristics and amount of chemicals being stored. The safety distance for the storage of 2000–3000 kg of flammable substances is 106 m; for more than 100,000 kg of flammable substances, it is 827 m. Even if the same flammable substance is present, the safety distance is 50 m when the flash point is less than 21 °C and 45 m when the flash point is between 21 °C and 70 °C. However, in Korea, the safety distances between plants are very small (less than 50 m) compared to those in China, and safety distances are not applied to chemicals from the US and EU [8]. The safety distance for flammable liquids is 30 m in Korea’s Occupational Safety and Health Act, 20 m in the Safety Control of Dangerous Substances Act, and 17 m in the Chemicals Control Act. This is very restrictive in comparison with other countries’ regulations, and safety distances are not subdivided by the type or amount of flammable liquids.

The purpose of this study was to examine the safety distance regulations in Korea compared to those of other countries, and to evaluate whether there is a possibility that a domino explosion will occur with the present safety distance regulations in Korea.

## 2. Materials and Methods 

### 2.1. Selection of Flammable Substances

The target substance in this study was a flammable liquid that could explode. Flammable liquids are defined as “hazardous materials dangerous in liquids” in the Safety Control of Dangerous Substances Act of the National Emergency Management Agency.

Even under the same flammable-liquids category, special phosphates, alcohols, 1 petroleum, 2 petroleum, 3 petroleum, 4 petroleum, and animal and plant oils are classified. Each division is specified according to the range of the flash point (e.g., 2 petroleum refers to kerosene, diesel, or other petroleum with a flash point of 21 °C to 70 °C at 1 atm) [9].

Korea is a country that has continuously developed through the petrochemical industry. Although it is not an oil-producing country, it has produced a variety of basic chemical products, such as refined crude oil, synthetic resin (plastic), and synthetic fibers (polyester and nylon). Among the manufacturing items in Korea, petrochemical products rank third, with exports of 36.2 billion dollar (7.3% of total exports) in 2017. Among petroleum products, ethylene, propylene, benzene, toluene, and xylene are the basic compounds being produced and exported. 

In particular, benzene, toluene, and xylene (BTX) account for 40% of the production of base oil. Unlike ethylene and propylene, the demand is also 90% of production [10]. BTX are also prescribed as flammable liquids in the Safety Control of Dangerous Substances Act [9]. We selected BTX as the substances to be studied in accordance with Korean regulations and industry trends. The physicochemical properties of the substances are shown in Table 1 [11].

### 2.2. Safety Distance Standard of Each Country

Currently, factories that store or handle flammable chemicals are required to maintain safety distances from other factory facilities. These safety distances are based on the distance between flammable facilities and vulnerable facilities (e.g., public facilities such as schools, hospitals, and cultural properties), which may affect the outside conditions when a fire or explosion occurs, and they should be apart. The safety distance varies depending on the country, and in the case of the US or EU, it may be classified according to the quantity and type of flammable substances to be handled and the type of business; the factory may also be moved in advance. These are specific regulations. On the other hand, countries such as Korea, Taiwan, or Dubai offer comprehensive safety distances regardless of the type of chemicals that they handle (e.g., 17 m from an outer wall and 50 m from a cultural property). These regulations are easy to maintain at the workplace, but may lead to secondary damage due to a fire or explosion if there is a chemical accident.

In China, a safety distance of 1000 m was set according to the Requirements and Technical Standards for Hazardous Chemical Firms after the explosion at Tianjin Port. If it can be verified that the facilities are safe, then it is reduced to 500 m. These safety distances can be considered overregulation. The rules for safety distances for each country are summarized in Table 2.

### 2.3. Domino Explosions Caused by Overpressure

Explosions may occur at workplaces handling flammable substances. When an explosion occurs, pressure is generated. The pressure that is delivered at the time of explosion that is at any point above atmospheric pressure is called overpressure. It is then possible to predict whether a domino explosion is likely to occur through the distance the overpressure reaches from the primary chemical facility. For example, when the distance over which an overpressure of 7 kPa (1 psi) reaches is 50 m, part of a house that is away from the site where the explosion occurred would be damaged. When typical overpressure is reached, the effect is summarized as follows [19] (Table 3).

If the overpressure reaches 24 kPa (3.5 psi), it will damage storage tanks. In this study, the standard explosion pressure at which a domino explosion could occur was 24 kPa (3.5 psi), and the distance that the overpressure of 24 kPa (3.5 psi) would reach when a chemical facility explodes was calculated. 

### 2.4. Vapor Cloud Explosion Method

A vapor cloud explosion (VCE) is caused by the continuous leakage of flammable hazardous substances in a container or piping, the gradual collection in the form of clouds in the atmosphere, and movement due to wind and convection. It is a phenomenon where all the gas explodes simultaneously because of the ignition source, such as exhaust gas. It causes significant damage because of the overpressure caused by the explosion. The methods of measuring the explosion are the trinitrotoluene (TNT) equivalence method, the Netherlands Organization (TNO) multi-energy method, and Baker-Strehlow-Tang (BST) method. 

#### 2.4.1. Trinitrotoluene Equivalence Method 

The TNT equivalence method is used to estimate overpressure assuming that the flammable energy in the vapor is converted to a TNT equivalent (1–10%). It is the simplest method and is widely used. However, the equivalence of TNT is not clearly defined, and the wave characteristics of TNT are different from those of some flammable liquids. In addition, when compared with measured values, overpressure values of explosion accidents caused by detonation are almost identical, but those of explosion accidents caused by deflagration are overestimated [19].

#### 2.4.2. The Netherlands Organization Multi-energy Method 

The TNO multi-energy method assumes that only the area occupied by the vapor cloud contributes to the storm (blast) when the explosion energy depends on the degree of gas concentration in the combustible area, and that the space is restricted because of space limitation or obstacles. It also has the concept of a single explosion that starts at one place and does not explode in series, but instead explodes in part of the vapor. It is assumed that the mass fraction of the combustible material in which the energy generated by the explosion increases does not have much effect on the explosion, but the degree of isolation of the generation region affects the explosive energy [20].

#### 2.4.3. Baker-Strehlow-Tang Method 

The Baker-Strehlow-Tang (BST) method is similar to the TNO multi-energy method. It assumes that the explosion will start in the part of the combustible vapor field where the process facilities are concentrated. An important point in this model is the flame speed. In the BST method, the TNO multi-energy method predicts the explosion intensity through the amount of combustible materials. However, the BST method predicts the direction of the flame and the presence of obstacles to predict the flame speed [21]. The core equation for each method and calculation flow chart are as follows [22]:

(1) $$\begin{array}{c} Z=\frac{L}{{\left(M_{TNT}\right)}^{1/3}}M_{TNT}=\frac{f_{E}\Delta H_{c}M_{G}}{\Delta H_{TNT}}\\ P_{s}=\frac{80,800\left[1+{\left(\frac{Z}{4.5}\right)}^{2}\right]}{\sqrt{1+{\left(\frac{Z}{0.048}\right)}^{2}}\sqrt{1+{\left(\frac{Z}{0.32}\right)}^{2}}\sqrt{1+{\left(\frac{Z}{1.35}\right)}^{2}}}\\ =Z\sqrt[{3}]{M_{TNT}}=Z\sqrt[{3}]{\frac{M_{G}f_{\varphi }\Delta H_{c}M_{G}\gamma }{\Delta H_{TNT}}} \end{array}$$

where *Z* is the scaled distance (m/kg^1/3^), *L* is the distance from the explosion center (m), *M_TNT_* is the amount of TNT (kg), *f*_E_: is the fraction of energy generated by the shock wave (0.01–0.1), $P_{s}$ is the overpressure (kPa), Δ*H_C_* is the combustion heat (kJ/kg), Δ*H_TNT_* is the combustion heat of TNT ($4.184\times 10^{6}\;J/\mathrm{kg}$), *M_G_* is the combustion heat of combustible gas (kg), *f* is the vaporization rate of the outflow gas, *ψ* is the explosion factor (usually 10% of the effluent gas contributed to the explosion), and *γ* is the ratio of TNT (the total energy of the mixed gas contributing to detonation and the ratio of the TNT equivalent energy to the explosion pressure generated).

Equation (1): TNT equivalence method equation:
Cloud dimensionsThe radius of the cloud (R) $R={\left(\frac{3\times V}{2\pi }\right)}^{1/3}$(2)Obstructed regionsa) X < 25 m b) X < 10 × D1 or X < 1.5 × D2Explosion blast strength (overpressure)$P_{s}^{\prime }=\frac{P_{s}}{P_{a}}$, $R^{\prime }=X\cdot {\left(\frac{E}{P_{a}}\right)}^{1/3}$ $P_{s}=10^{-b\cdot (\log_{10}(r^{\prime }-c)}$Positive phase duration$t_{p}=\left(\frac{t_{p}^{\prime }}{C_{s}}\right)\cdot {\left(\frac{E}{P_{a}}\right)}^{1/3}$
where P_s_ is the overpressure caused by the explosion (MPa), $P_{s}^{\prime }$ is the converted overpressure (MPa), $R^{\prime }$ is the conversion distance (m), $P_{a}$ is the atmospheric pressure (MPa), X is the distance from the explosion (m), and E is the emission energy from the explosion (MJ).

Equation (2): TNO multi-energy method calculation flow chart.
Cloud dimensionsThe radius of the cloud (R) $R={\left(\frac{3\times V}{2\pi }\right)}^{1/3}$(3)Flame speedFlame expands: classified into three categoriesExplosion blast strength (overpressure)Flame speed in Mach numbers

Equation (3): BST method calculation flow chart.

### 2.5. Setting Modeling Conditions

To proceed with modeling, the legal basis of the capacity of the flammable liquid storage tank was as follows. The amount of flammable material in the regulations for safety distance of flammable material in England is specified as a range from 0.1 kg to 100,000 kg [16]. In Taiwan, the capacity of flammable materials is specified as from 2000 kg to 200,000 kg [18]. In the US, it is defined as 1000 kg to 1,000,000 kg [15]. According to the Safety Control of Dangerous Substances Act of Korea, regulations start from at least 100,000 kg for the designated quantity of flammable liquid [23]. In particular, the size of BTX storage tanks in Korea in 2018 was between 20,000kg to 50,000 kg. [24]. Therefore, in this study, it was assumed that the accident occurred on the basis of 50,000 kg in order to estimate the simulation of a flammable liquid storage tank as conservatively as possible. The conditions underlying the model are shown in Table 4.

The atmospheric stability of the meteorological condition in the explosion model was calculated as D grade. These meteorological conditions are those applied when modeling the worst-case scenario. This conservatively estimated the conditions of the atmosphere and did not rule out the possibility of a domino explosion. The table for Pasquil’s atmospheric stability is shown below [25] (Table 5).

### 2.6. Modeling Tool Selection

There are various modeling tools for estimating VCE. However, in this study, we selected the tool based on each methodology and compared the results with the safety distance. 

The TNT equivalence method was modeled by direct computation. This was because it is the simplest and is used for explosion prediction in industrial fields.

Based on the TNO multi-energy method, Process Hazard Analysis Software Tool (PHAST) was selected as the modeling tool. PHAST is a commercial program developed by Det Norske Veritas, which is a German-Dutch joint venture. The TNO and BST methods can be used to model the program, but in this study, the TNO method was used. Finally, the tool used by the BST method was Areal Location of Hazardous Atmospheres (ALOHA), which is supported by the US EPA. ALOHA was developed based on the BST method in VCE modeling and has a disadvantage in that it does not support other methodologies [26].

## 3. Results

### 3.1. Overpressure Distance Comparison

The range that the overpressure reached when the VCE occurred was directly calculated and modeled using a modeling tool. Even if all 50,000 kg of flammable liquid were leaked, the whole volume would not be converted into steam and, subsequently, explode. After it forms a pool on the ground, it evaporates and becomes explosive. In other words, accidents involving steam explosions would occur under conditions that continuously leak and form steam. The amount of vaporized substance was estimated using a tool (PHAST). In the TNT equivalence method, the heat of combustion (kJ/kg) for each substance and the amount of leakage (kg) that could be generated by evaporation were calculated.

**Figure 1:**
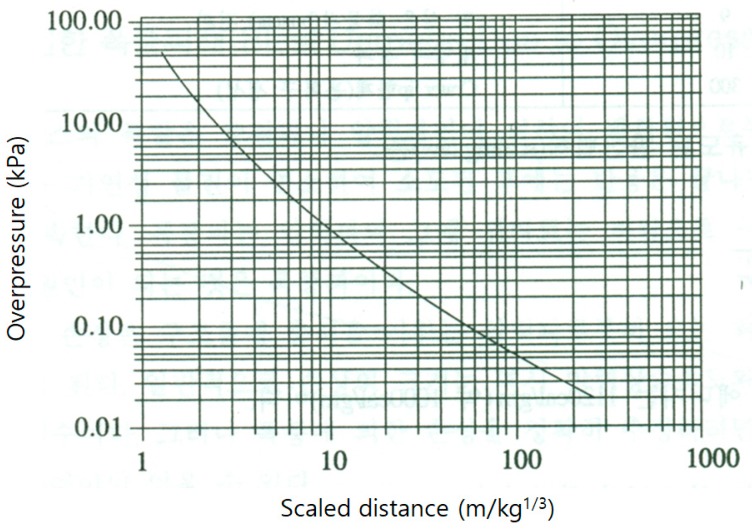
Relationship between scaled distance and overpressure (trinitrotoluene).

Since TNT has enough experimental data for each explosion pressure, it was possible to deduce the magnitude of the overpressure using the scaled distance. By reversing the *Z* value in Equation (1), the distance *L* (m) from the explosion center that the desired overpressure reached could be derived. In particular, this continuous calculation could be used to infer and trace back the conversion distance curve when each chemical explodes. Figure 1 shows the relationship between the scaled distance and the overpressure for TNT.

**Figure 2:**
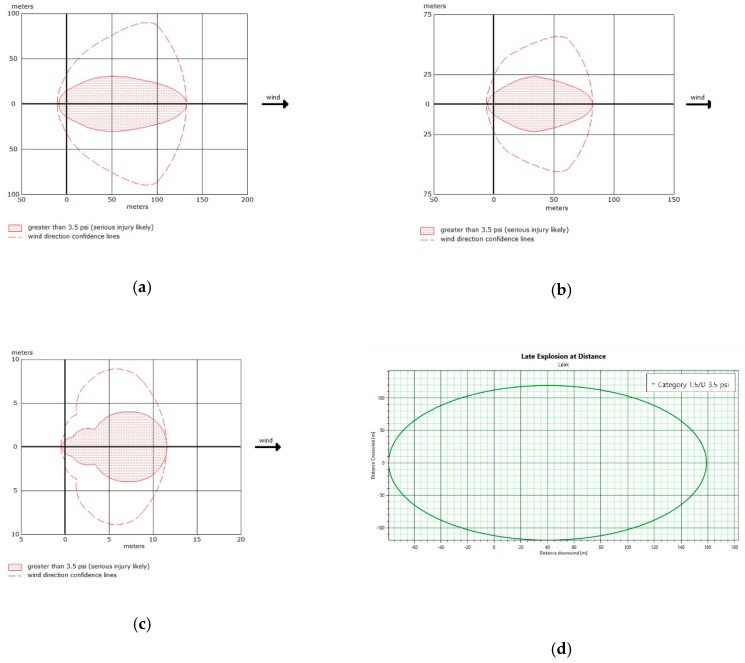

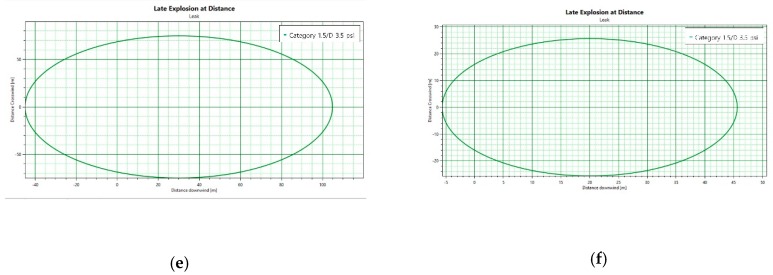
Overpressure distance graph (**a**) BST method for benzene 133 m (**b**) BST method for toluene 86 m (**c**) BST method for xylene 13 m(**d**) TNO multi-energy method for benzene 161 m (**e**) TNO multi-energy method for toluene 102 m (**f**) TNO multi-energy method for xylene 45 m.

The overpressure at which neighboring chemical facility storage tanks could be destroyed was 24 kPa (3.5 psi), but the distance with 7 kPa (1 psi) was also calculated for comparison. The results are shown in Table 6 and. Figure 2 shows the result of modeling with TNO multi-energy method and BST method. 

### 3.2. Safety Distance Comparison

The safety distance could be compared to determine if there is a potential for domino explosions in each country. The table was based on 50,000 kg of flammable materials stored in a factory with outdoor storage tanks. If the safety distance is longer than the distance over which the overpressure of 24 kPa (3.5 psi) can reach, then the probability of a domino explosion is low. However, if the safety distance is shorter than the distance that the overpressure can reach, then the chemical equipment inside this distance will suffer secondary damage from the domino explosion. There was no significant difference between the distance that the overpressure reached with each method. This was because each method is based on the TNT method. The overpressure distance was applied as the average value of the three methods, and the safety distances of the countries were compared, as shown in Table 7.

### 3.3. Case Study 

In Korean industries, major petrochemical plants manufacture BTX or chemical production plants produce products using BTX as raw materials. In addition to BTX, petrochemical manufacturers handle many hazardous chemicals, including flammable gases, and there are many techniques for securing safety in the chemical industry. However, manufacturers who treat BTX as a raw material (paint manufacturing, plastic-related product manufacturing, etc.) are relatively vulnerable to chemical accidents [27].

In this study, we presented a domino explosion simulation and its results for BTX-related solvent chemicals of paint manufacturers.

In addition to toluene and xylene, the simulated plant also handles large quantities of flammable substances, such as butyl acetate and styrene, and stores them in 50-ton storage tanks outdoors. These solvents are most often used as raw materials in the manufacturing of paints, and the manufactured products are stored separately in storage warehouses. However, since they are flammable substances, fire accidents may occur. These solvents are stored 10 storage tanks, and a tank farm is created and stored in one place for convenience. 

If the flammable liquid storage tank begins to leak and an explosion occurs, then the primary explosion will not be over. In the simulation, when the first explosion occurred in toluene storage tank 1 inside the factory at the tank farm, a maximum overpressure of 24 kPa (3.5 psi) was reached at a radius of 108 m. Second, a 26 m explosion took place in a xylene tank 3 m from toluene tank 1. The same overpressure was also reached at toluene storage tank 2, which was 70 m from the third tank, thereby resulting in an explosion. The explosion of toluene tank 2 affected plant B. Plant B was in accordance with the Korean safety distance and was moved in accordance with the 20 m separation distance. However, the explosion range of toluene tank 2 affected the chemical storage room of factory B. The simple domino explosion did not end inside plant A, but affected the nearby plant B as well.

**Figure 3:**
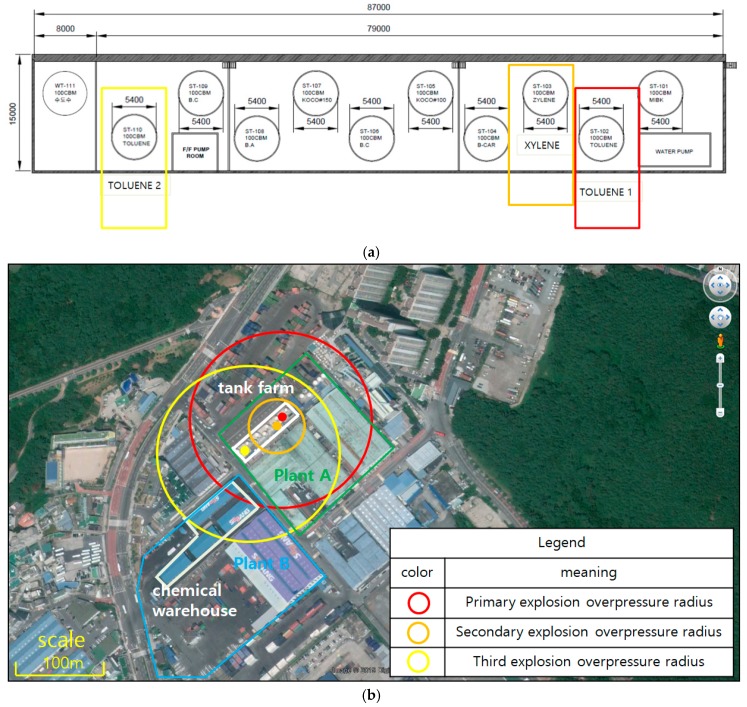
(**a**) Tank farm layout and (**b**) domino effect explosion scenario.

In Korea, chemical manufacturers of similar industries are concentrated in the same region. In the simulation, there is also a paint factory in plant B near plant A, and a chemical storage warehouse. In this case, local chemical accidents may occur, such as the explosion in Tenjin Port, rather than an accident that only ends in one plant. Figure 3 shows the tank layout of plant A’s tank farm and the damage when the domino explosion occurred.

## 4. Discussion

In this study, the safety distance of the chemical storage facility was properly regulated. The safety distance of chemicals is important because it is the most important way to prevent secondary and tertiary damage caused by the primary chemical accident. At each factory, various safety devices are used to prevent chemical accidents. This, in addition to installing additional safety devices (e.g., sprinklers, gas detectors, and personal protective equipment to prevent fires), can prevent chemical accidents through initial factory location analysis.

It is difficult to change the location of factories in Korea’s industrial structure. However, this study shows that Korean regulations should be changed in comparison with foreign safety distance regulations.

Chemical accidents caused by simple leaks and the spread of toxic substances can prevent initial response and diffusion. This means that there is time to respond early in an accident such as a leak. However, since the explosion is an accident that occurs immediately, it is difficult to cope with the initial explosion. Safety distances are therefore important to prevent explosion accidents. 

In particular, BTX materials, which are used domestically and are the basis of the chemical industry, are modeled and confirmed the required safety distance.

Modeling results and actual chemical accidents may be different. The actual explosion may be smaller than that in the model or result in a fire without an explosion. However, accident prevention should be approached from a more conservative perspective. Benzene and toluene can cause an overpressure that can reach over 100 m. Xylene can cause an overpressure that can reach a relatively short distance of 26 m. This is because of the physicochemical properties of xylene itself. Xylene has a lower vapor pressure (benzene: 10 kPa, toluene: 5 kPa; xylene: 2 kPa) [11]. The flash points of benzene and toluene are lower than 21 °C, which is Flammability Class 4 of the NFPA 704 code (Standard System for the Identification of Materials for Emergency), and xylene is in Class 3 [28]. For these reasons, it is presumed that the overpressure level of xylene is small. It can be seen that a safety distance of 100 m or more is required when a relatively flammable and explosive substance is used. On the other hand, among the same flammable substances, the safety distance of less than 30 m is safe for chemicals with low flammability class. Safety distances shall be considered according to the nature of the material.

There are three methods for explosion modeling. There was no significant difference between the methods. It is possible to calculate without a separate tool, and values can be derived more easily if the physicochemical characteristics of the chemical to be handled are known. However, in the case of flammable liquids, the three methods had similar results, even though previous studies showed that they can be different for flammable gases. In the case of flammable gas leaks, the amount of storm clouds that can cause an explosion depends on the state of the atmosphere or the type of accident. For example, windy environments can spread or dilute the gas as it leaks, thereby reducing the likelihood of an explosion. In contrast, if a large amount of gas leaks at once, then there is a possibility that a large explosion will occur instantaneously. In particular, some reports have stated that the TNT method provides an underestimation compared to the actual accident, so it can be problematic to use only one methodology. Conversely, the TNO method or BST method may yield exaggerated results. Continuous research is needed for flammable gases [22].

The safety distances of each country are larger than those of Korea, Taiwan, and Dubai. Especially in England, the safety distance is suggested according to the storage amount of various flammable substances. The safety distance between not only simple chemical facilities, but also highways, buildings, railroads, and general commercial stores are detailed. Other countries besides England in the EU also submit information to the state through modeling or calculations, and are authorized by the workplace in accordance with the Seveso Directive to locate facilities in consideration of domino explosions. The US offers safety distances for each industry. It varies from a minimum of 100 m to 1000 m. Even in the same industry, distances vary depending on the amount of flammable substances being handled.

In contrast, Korea has no regulations or guidelines related to domino explosions. However, there is a slight difference between the Occupational Safety and Health Act, Safety Control of Dangerous Substances Act, and Chemicals Control Act, so it is necessary to review the uniformity and effectiveness of laws and regulations. In Taiwan, the safety distance is divided by the range depending on the flash point of the flammable substance. However, the maximum safety distance is only 40 m. In this case, a flammable substance with a flash point of less than 21 °C is stored in quantities exceeding 400,000 kg. Considering the result that the overpressure range of 50,000 kg of flammable material is more than 100 m, it is necessary to subdivide and modify the regulation. 

The case study showed the problems of Korean industrial complexes and the possibility of domino explosions. In particular, plants A and B were located at a safe distance of 20 m. Flammable liquids are stored in a large dike which is about 87 m wide and 15 m long, but there was no breach in Korean chemical management regulations. However, the explosion of flammable liquid may cause damage to other plants through secondary explosions. If an explosion or fire spreads to another neighboring plant, then it can further spread to other nearby areas.

This case satisfied all laws and regulations related to companies handling chemical substances. However, if an explosion occurred, a second and third domino explosion would be likely, regardless of the regulations. Countries are urged to prevent chemical accidents by imposing rules on safety distances.

## 5. Conclusions

The purpose of this study was to investigate the possibility of secondary and tertiary domino explosion accidents caused by an initial chemical accident not only in the first chemical accident, but also in the case of chemical substances that could cause a fire or explosion. The study examined the appropriateness of safety distance regulations.

Safety distance is the distance that must be maintained between chemical facilities or between chemical facilities and other buildings (schools, roads, hospitals, etc.).

The legal restrictions on safety distance vary greatly by country. Assuming that BTX (benzene, toluene, and xylene), i.e., common flammable substances in the workplace, are stored in a 50,000 kg storage tank, Korea is above the safety distance as a result of the explosion accident simulation. The theory of the VCE proceeded with three methods, namely the TNT equivalence method, TNO multi-energy method, and BST method. As a result, an overpressure of 24 kPa (3.5 psi) reached an average distance of 146 m for benzene, 108 m for toluene, and 26 m for xylene. A force of 24 kPa (3.5 psi) can destroy storage tanks and disrupt the steel structure. That is, other flammable material storage tanks within the overpressure range of the flammable material may also explode.

Korea is particularly vulnerable because the regulations on safety distances are not as subdivided as those in the US or England, and there are no provisions related to domino explosions. Since regulations in the US and England are subdivided and safety distances are relatively large (300 m to 1000 m), the possibility of a domino explosion is low. In the case of China, the possibility is low because it is tightly regulated.

However, countries such as Korea, Taiwan, and Dubai have safety distances of only 20 m to 30 m. Therefore, there is a possibility that domino explosions may occur at plants storing large amounts of flammable substances. However, explosion simulations differ significantly depending on the physicochemical properties of the flammable substances, the amount handled, and the storage conditions and processes.

Based on the results of this study, BTX plants in Korea should consider distances from neighboring chemical compounds of 150 m for benzene, 120 m for toluene and 30 m for xylene in order to avoid domino explosions. Therefore, it is necessary to propose a reasonable method to determine safety distances considering the physicochemical characteristics and amount of chemical substances being handled to prevent the occurrence of domino explosions.

## Figures and Tables

**Table 1:** Physicochemical properties of benzene, toluene, and xylene.

Chemical Name	Benzene	Toluene	Xylene
CAS No.	71-43-2	108-88-3	1330-20-7
Molecular Formula	C_6_H_6_	C_7_H_8_	C_8_H_10_
Molecular Weight	78.11	92.14	106.16
Flash Point (°C)	−11 (closed cup)	4 (closed cup) 16 (open cup)	17 (closed cup) ^1^ 25 (closed cup) ^2,3^
Ignition Temperature	580	480	463 ^1^; 528 ^2^; 527 ^3^
Explosion Limit (%)	1.2–7.8	1.27–7.80	0.9–6.7 ^1^ 1.1–7.0 ^2,3^

^1^*o*-xylene; ^2^*m*-xylene; ^3^*p*-xylene.

**Table 2:** Safety distance standard and summary in each country.

Country	Safety Distance (m)	Set Criteria Basis
Korea	20	Occupational Safety and Health Act (20 m) and Safety Control of Dangerous Substances Act (10 m) [12] Chemicals Control Act: 30 m for a cultural facility or a meeting facility with 300 or more people (e.g., school, movie theater, etc.) and 10 m between industrial facilities [13]
China	1000	Article 6 of the Requirements and Technical Standards for Hazardous Chemical Firms: 500 m if safety measures are recognized [14]
US	300–1000	Separation Distances Between Industrial and Sensitive Land Uses: varies depending on the storage amount of industrial and flammable substances [15]
England	18–500	Explosives Regulations Health and Safety Executive: safety distance must be presented through modeling for each workplace [16]
Dubai	11 ^1^ 15 ^2^	Regulation DD-19.0: Hazardous/Dangerous Chemical Storage Warehouse [17] ^1^ 11 m for indoor chemical facilities ^2^ 15 m for outdoor chemical facilities
Taiwan	20	Public Hazardous Substances & Flammable Pressurized Gases Establishment Standards & Safety Control Regulations [18]: 20 m, but if the flash point is higher than 21 °C, an additional 1 m will be added and 2 m will be added when storing more than 2000 kg

**Table 3:** Determination of the influence of explosion overpressure.

Overpressure		Effect
kPa	Psi	
0.15	0.02	Noise generation
0.2	0.03	Partial breakage of windows
1	0.15	Glass rupture pressure
2	0.3	10 % damage to the roof and windows of the house
5	0.7	Structural damage of houses
7	1	Some damage to the house (unrecoverable)
15	2	The wall and roof of the house are slightly damaged
20	3	The steel structure of the building is damaged and deviates from the foundation
24	3.5	Damage to steel structures or storage tanks
30	4	Damage to factory buildings
35–50	5–7	Complete destruction of the house
50–55	7–8	Brick wall with a thickness of 20–30 cm collapses
60	9	Total breakage of large heavy lorry
70	10	Most buildings destroyed

**Table 4:** Modeling conditions.

Type of Storage Tank	Vertical Tank
Storage tank size	2 m ^1^ and 4 m ^2^
Size of piping (diameter of orifice)	50 mm
Air temperature (°C), wind speed (m/s), and humidity (%) during leak	25, 1.5, and 50
Atmospheric stability of Pasquil	D
Standard overpressure	24kPa (3.5 psi)

^1^ Radius; ^2^ Height.

**Table 5:** Pasquil atmospheric stability.

Wind Speed (m/s)	Day			Night	
	Radiation Intensity				
	Strong	Moderate	Slight	Cloudy	Sunny
<2	A	A–B	B	F	F
2–3	A–B	B	C	E	F
3–5	B	B–C	C	D	E
5–6	C	C–D	D	D	D
>6	C	D	D	D	D

**Table 6:** Overpressure distance comparison.

Method	Overpressure (kPa)	Overpressure Distance (m)		
		Benzene	Toluene	Xylene
TNT equivalence method	24	144	138	22
	7	272	252	42
TNO multi-energy method	24	161	102	45
	7	243	171	76
BST method	24	133	86	13
	7	217	123	25

**Table 7:** Comparison of the possibility of a domino explosion in each country.

Country	Safety Distance (m)	Benzene Overpressure Distance (146 m)	Toluene Overpressure Distance (108 m)	Xylene Overpressure Distance (26 m)
Korea	20	+ ^1^	+ ^1^	+ ^1^
China	1000	- ^2^	- ^2^	- ^2^
US	1000	- ^2^	- ^2^	- ^2^
England	877	- ^2^	- ^2^	- ^2^
Dubai	15	+ ^1^	+ ^1^	+ ^1^
Taiwan	30	+ ^1^	+ ^1^	-

^1^ Possible domino explosion; ^2^ Domino explosion is unlikely.
